# The effect of exercise intervention on amyotrophic lateral sclerosis: a systematic review and meta-analysis

**DOI:** 10.3389/fneur.2025.1499407

**Published:** 2025-05-21

**Authors:** Shuangquan Ren, Xinrui Che, Shunding Hu, Xiaosu Feng, Jianming Zhang, Peng Shi

**Affiliations:** ^1^Northeast Petroleum University, Daqing, China; ^2^Liaoning Police College, Dalian, China; ^3^School of Physical Education, Liaoning Normal University, Dalian, China; ^4^School of Physical Education, Shandong University of Technology, Zibo, China

**Keywords:** amyotrophic lateral sclerosis, physical function, respiratory function, exercise, meta-analysis

## Abstract

**Objective:**

Quantitative evaluation of the effect of exercise intervention in amyotrophic lateral sclerosis (ALS).

**Methods:**

The CNKI, WOS, PubMed, and Scopus databases were searched by computer, and randomized controlled trials (RCTs) of exercise intervention in ALS were screened out according to the inclusion and exclusion criteria of the PICOS principle. Stata 12.0 software was used for statistical analysis.

**Results:**

A total of 12 RCTs including 430 participants were included. Meta-analysis results show that exercise intervention can significantly improve the overall function, walking test (WT) distance and maximum expiratory pressure (MEP) of ALS patients (*p* < 0.05). However, exercise interventions did not show significant effects on fatigue, maximum inspiratory pressure (MIP), forced vital capacity (FVC), and peak expiratory flow (PEF) in ALS patients (*p* > 0.05). Subgroup analysis showed that resistance exercise is the most effective intervention for improving the function of ALS patients, while aerobic exercise is the most effective intervention for improving FVC in ALS patients.

**Conclusion:**

Exercise intervention in ALS has a positive effect, but due to the small number of included studies and possible heterogeneity, risk of bias and sensitivity issues, further research is needed.

## 1 Introduction

Amyotrophic lateral sclerosis (ALS) is a progressive, lethal motor neuron disease. It usually involves upper motor neurons (brain, brainstem, spinal cord) and lower motor neurons (cranial nerve nuclei, anterior horn cells of the spinal cord) and manifests mainly as muscle weakness, atrophy, dysarthria and dysphagia (1, 2). ALS most often develops between the ages of 50 and 60 and most patients die within 3–5 years from respiratory paralysis or lung infection (3).

Although ALS is still a disease that cannot be completely cured at present, there are many ways to improve patients’ quality of life and slow down disease progression, such as nutritional management (4), assisted breathing (5), psychotherapy (6) and medication (7). However, the aforementioned strategies may have some limitations, such as being highly passive, causing side effects, and having limited functional improvement (1, 8). For example, assisted ventilation typically relies on devices such as ventilators to help patients breathe, which restricts their range of activities and independence in daily life (1). In addition, pharmacological treatments often come with side effects such as nausea, vomiting, and dizziness, and long-term use may potentially cause damage to liver and kidney functions (9). Moreover, the above treatments mainly focus on alleviating local symptoms in ALS patients. For instance, nutritional management emphasizes providing the necessary material basis for the body, assisted ventilation targets only respiratory function support, and psychological therapy primarily aims to adjust the patient’s psychological state and alleviate emotional issues to improve quality of life. However, these approaches have insufficient impact on enhancing the patients’ overall physical condition, improving daily physiological functions, and ultimately, on improving quality of life.

As research continues to advance, moderate exercise tailored to the patient’s disease stage and physical condition is considered beneficial for ALS patients (10). For example, strength exercises to slow down weakness, flexibility exercises to reduce contractures, and prolonged activities such as walking to maintain endurance (11). Patients with ALS are often physically weak and frequently experience complications such as muscle atrophy, muscle spasms, respiratory difficulties, and sensory abnormalities (12, 13). Therefore, traditional medical advice for ALS patients usually emphasizes avoidance of exercise to prevent injury and exacerbation of the condition due to exercise (14, 15). However, most studies (16, 17) recommend that patients with neuromuscular diseases engage in moderate exercise tailored to their condition to maintain physical function and improve quality of life. The reason is that increasing the content of mitochondria in the muscles can enhance blood flow to the muscles, thereby maintaining and enhancing muscle strength, which in turn helps to maintain their ability to perform daily activities (16, 17). Therefore, the results of current studies on the effects of exercise on ALS patients and the relationship between the two are inconsistent. In addition, studies by Kilmer (14) and Francis et al. (18) have concluded that the effect of exercise on ALS patients is controversial.

In fact, studies (19–21) have already used systematic reviews and meta-analyses to explore the effects of exercise interventions on ALS. However, these studies included a variety of exercise types, such as aerobic exercise, resistance exercise, combined exercise, and respiratory muscle training. Yet, they fell short in investigating which type of exercise is more beneficial for ALS patients. In other words, the relationship between exercise type and outcome measures is unclear, and it is uncertain which type of exercise can better promote the improvement of specific indicators. Therefore, they are unable to provide a more precise exercise guideline for ALS patients. Therefore, this study systematically searched for studies related to exercise interventions for ALS, and explored the effects of exercise interventions for ALS through systematic review and meta-analysis. Additionally, subgroup analyses were conducted to explore the effects of different types of exercise interventions and to clarify the associations between exercise types and outcome measures in ALS patients. This study is used to facilitate health care workers to understand the risks and benefits of exercise in order to develop a safe and appropriate exercise plan.

## 2 Methods

The study was written and reported in strict accordance with the PRISMA 2020 entry list (22) for the search strategy, selection criteria, data extraction, bias assessment and mathematical statistics.

### 2.1 Search strategy

One researcher conducted the relevant literature search in English and Chinese. The search terms “amyotrophic lateral sclerosis (ALS),” “motor neuron disease (MND)” and “Lou Gehrig” were combined with “sport,” “exercise,” “training” and “fitness” in CNKI, Web of Science (WOS), PubMed and Scopus. The search time frame is from the creation of this database to November 2022.

### 2.2 Selection criteria

This study was designed with inclusion and exclusion criteria based on the PICOS principles (23). Inclusion criteria: (1) The subjects were patients with ALS; (2) The trial measures were any forms of exercise intervention; (3) The control measures included daily activities, usual care, stretching exercises, neuro-rehabilitation therapy and placebo exercise; (4) The outcome variables included at least one of ALS function, fatigue, WT (walk test), MIP (maximum inspiratory pressure), MEP (maximum expiratory pressure), FVC (forced vital capacity), PEF (peak expiratory flow rate); (5) The study design was a randomized controlled trial (RCT). Exclusion criteria: (1) Animal experiments, case reports, single-group experiments, quasi-experimental designs; (2) Positive controls in multi-arm studies; (3) Reviews, abstracts, letters, commentaries; (4) Incomplete data information on outcome variables; (5) Combined interventions that include exercise, such as the combination of exercise and nutrition; (6) Repeated publications for the same subjects, including only literature of relatively high quality. The selection process follows the order of title, abstract, figure, and full text. The literature was screened by two researchers independently according to the selection criteria, and the screened literature was secondarily assessed by two other researchers, and if there was a dispute, it was mutually agreed in a group discussion.

### 2.3 Data extraction

The data extract includes the first author, publication date, study design, participant characteristics, exercise protocol and outcome variables. For this study, the extracts were entered into Excel 2010 and saved. The data extraction was carried out independently by two researchers, and the extraction was secondarily assessed by two other researchers, and if there were controversial issues, a group discussion was held to decide jointly.

### 2.4 Quality assessment

This study used the risk of bias assessment tool recommended by the Cochrane Collaboration Network (24, 25) to assess the risk of bias for RCTs. The tool assesses six aspects of random methods, blinding, allocation concealment, completeness of outcome data, selective reporting of findings and other biases. Two researchers judged independently on the basis of the assessment tool, and where there were serious disagreements, the entries were discussed with a third researcher.

### 2.5 Data analysis

This study used Stata 12.0 software for data processing and statistical analysis. The models were selected by heterogeneity tests to test for combined effects. The *Q*-test and *I^2^* statistic were used to test for between-study heterogeneity. If *I^2^* < 50% and *p* > 0.1, the between-study heterogeneity was considered small and a fixed-effects model was selected for analysis; if *I^2^* ≥ 50% and *p* ≤ 0.1, the between-study heterogeneity was considered large and a random-effects model was selected for analysis. This study explores the effects of different exercise types on ALS through subgroup analysis. This study explored the sources of heterogeneity through one-way meta-regression analysis. The study was tested for publication bias through Egger linear regression analysis. Sensitivity analysis was conducted using the “metainf” command for the one-by-one elimination method. Effect sizes for continuous variable information are expressed using standardized mean differences (SMD) and 95% confidence intervals (CI) are used to express the estimated intervals of the overall parameters constructed from the sample statistics. The level of heterogeneity was set at *α* = 0.1 and the rest of the tests at *α* = 0.05.

## 3 Results

### 3.1 Selection results

**Figure 1:**
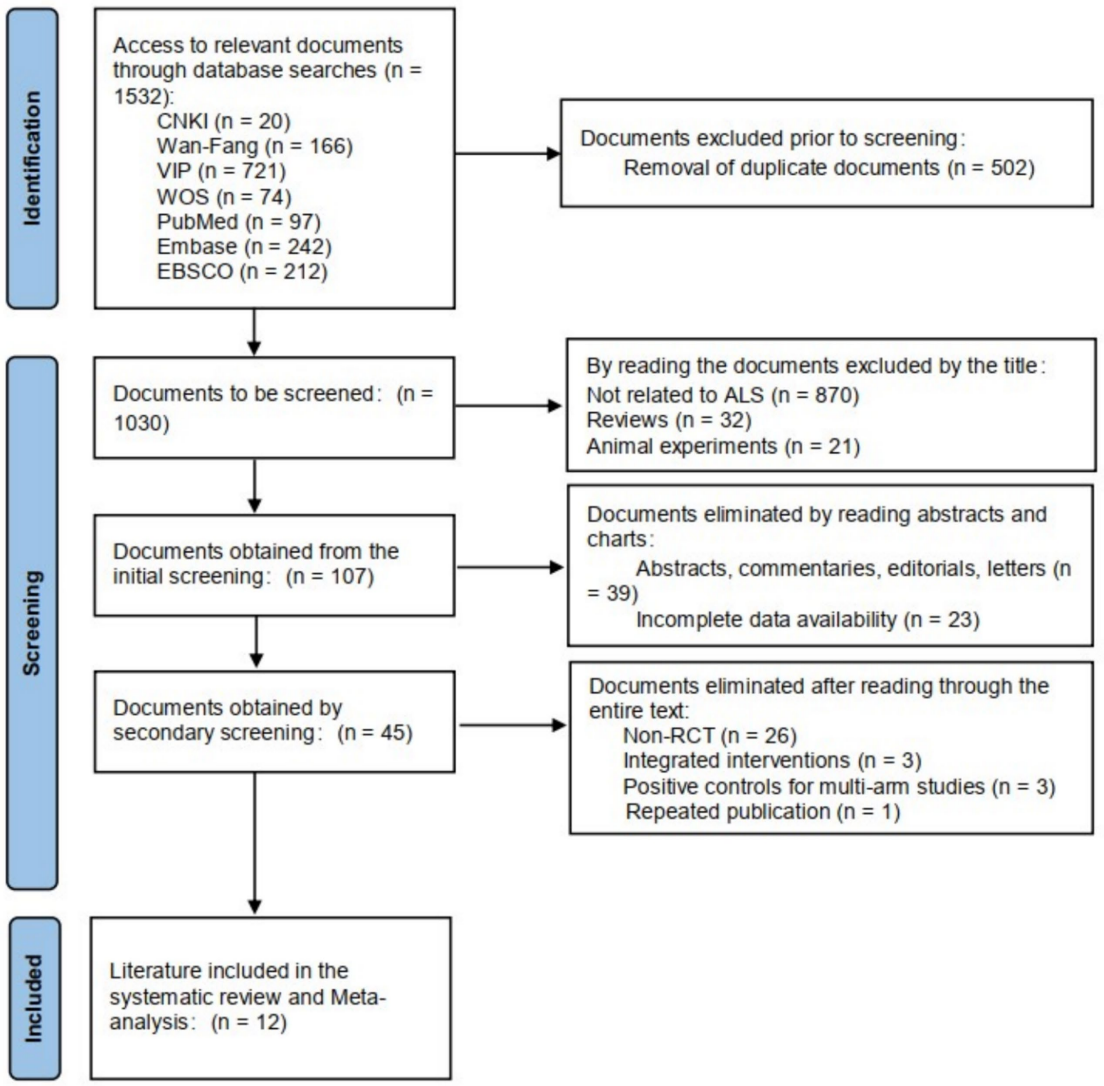
Flow chart for literature screening.

In this study, 238 documents were retrieved, including 20 Chinese documents and 218 English documents. The retrieved literature was imported into EndNote X9 software for de-duplication, resulting in 160 documents. A total of 12 documents were selected for inclusion. The literature screening process is shown in Figure 1.

### 3.2 Data extraction results

Publication dates for the included studies were 2001 (26) to 2021 (27). A total of 430 participants were included in the meta-analysis to explore the effects of exercise interventions to improve ALS, including 222 in the test group and 208 in the control group. The mean age of the test group was 50.7 (28) ~ 63.2 (11) years old and the mean age of the control group was 53.4 (29) ~ 62.1 (30) years old. The proportion of females in the test group was 25.0% (11, 28) ~ 61.1% (27) and 25.0% (28) ~53.3% (30) of females in the control group. Participants were obtained from eight countries: Canada (31), Israeli (26, 27), Italy (32, 33), Australia (28, 29), United Kingdom (34), United States (30, 35), Portugal (11), and the Netherlands (36), of which studies with samples of ALS patients from Israel, Italy, Australia, and the United States included two each. Six studies (11, 26, 31–33) used the ALS Functional Scale (ALSFRS) as the standard for clinical judgment of ALS patients; five studies (27, 28, 30, 35, 36) based the diagnosis on the revised El-Escorial criteria; and the remaining one study (29) only indicated that the enrolled ALS patients had a clinical diagnosis, without mentioning the specific criteria. The exercise intervention program in the test group consisted of four types of aerobic exercise (11), resistance exercise (26, 31, 33), combined exercise (27, 28, 32, 36), and respiratory muscle training (29, 30, 34, 35). The exercise period was 5 weeks (32) ~52 weeks (26), the frequency of exercise was 2 times/week (26, 34) ~21 times/week (29), and the duration of each session was 10 min (29, 34) ~60 min (32). The participants in the control group mainly performed stretching exercises (27, 31), daily activities (26, 28), nursing training (11), and so on. All included studies collected relevant data immediately after the intervention. Among them, 11 studies (11, 26–28, 30–36) assessed the ALS function of the participants; 6 studies (26, 27, 31, 32, 34, 36) evaluated the fatigue level of the participants; 2 studies (27, 32) assessed the walking ability of the participants; 3 studies (27, 29, 34) evaluated the maximum inspiratory pressure of the participants; 7 studies (11, 27, 29, 33–36) assessed the forced vital capacity of the participants; 2 studies (34, 35) evaluated the peak expiratory flow rate of the participants; 2 studies (27, 35) assessed the maximum expiratory pressure of the participants. In addition, no studies reported adverse reactions among the participants. The basic characteristics of the included studies are shown in Table 1.

**Table 1:** Basic characteristics of the included documents.

Included studies	Subject characteristics (*n*/ age/ F%/ nationality/ clinical information)		Exercise protocol		Outcome variables		
	TG	CG	TG	CG	Time points	Variables	Adverse reactions
Bello-Haas et al. (2007) (31)	*n* = 11 Age = N/A F% = N/A Canada ALSFRS≥30	*n* = 14 Age = N/A F% = N/A Canada ALSFRS≥30	6 months resistance exercise + stretching (3 times/day)	Stretching exercises (1 time/day)	Immediately after the intervention	①②	No
Drory et al. (2001) (26)	*n* = 14 Age = 60 (41 ~ 80) F% = 42.9% Israeli Mean ALSFRS = 27.5	*n* = 11 Age = 60 (41 ~ 80) F% = 45.5% Israeli Mean ALSFRS = 27.5	12 months moderate intensity muscle workout (2 times/day, 15 min/time)	Daily activities	Immediately after the intervention	①②	No
Merico et al. (2018) (32)	*n* = 23 Age = 61.6 ± 10.6 F% = 43.5% Italy Mean ALSFRS = 36.1 ± 4.71	*n* = 15 Age = 59.8 ± 14.7 F% = 26.7% Italy Mean ALSFRS = 34.5 ± 3.6	5 weeks aerobic exercise (65% HRmax, 5 times/week, 15-20 min/time) combined with resistance exercise (3 repetitions, 30s interval) for 1 h	Neuro-motor rehabilitation	Immediately after the intervention	①②③	No
Cheah et al. (2009) (29)	*n* = 9 Age = 54.2 ± 9.8 F% = 33.3% Australia Clinical diagnosis	*n* = 10 Age = 53.4 ± 9.5 F% = 40.0% Australia Clinical diagnosis	12 weeks IMT (15 to 60% SNIP, 7 days/week, 3 times/day, 10 min/time)	Placebo	Immediately after the intervention	④⑤	No
Pinto et al. (2012) (34)	*n* = 13 Age = 57.1 ± 9.3 F% = 46.2% United Kingdom ALSFRS = 24/40	*n* = 13 Age = 56.8 ± 8.7 F% = 15.4% United Kingdom ALSFRS = 24/40	8 months IMT (30% MIP, 2 times/day, 10 min/time)	Placebo	Immediately after the intervention	①②④⑤⑥	No
Plowman et al. (2016) (30)	*n* = 25 Age = 62.2 ± 10.5 F% = 44.0% United States Diagnosis based on the revised El-Escorial criteria	*n* = 15 Age = 62.1 ± 13.2 F% = 53.3% United States Diagnosis based on the revised El-Escorial criteria	5 weeks EMST (50% MEP, 5 days/week, 20 min/day)	Placebo	Immediately after the intervention	①	No
Plowman et al. (2019) (35)	*n* = 24 Age = 63.1 ± 10.1 F% = 29.2% United States Diagnosis based on the revised El-Escorial criteria	*n* = 24 Age = 60.1 ± 10.3 F% = 50.0% United States Diagnosis based on the revised El-Escorial criteria	8 weeks EMST (50% MEP, 5 days/week, 20 min/day)	Placebo	Immediately after the intervention	①⑤⑥⑦	No
Lunetta et al. (2016) (33)	*n* = 30 Age = 61.1 ± 10.1 F% = 30.0% Italy ALSFRS-R = 39.1 ± 4.7	*n* = 30 Age = 60.3 ± 9.9 F% = 43.3% Italy ALSFRS-R = 38.3 ± 5.1	6 months resistance exercise (60% MPO, 3 reps/set for 20 min)	Routine care	Immediately after the intervention	①⑤	No
Braga et al. (2018) (11)	*n* = 24 Age = 63.2 ± 13.0 F% = 25.0% Portugal ALSFRS-R ≥ 30	*n* = 24 Age = 62.0 ± 12.1 F% = 41.0% Portugal ALSFRS-R ≥ 30	6 months moderate intensity aerobic exercise (20% CEPT, 2 times/week) + nursing training (exercise routine and gait exercises)	Nursing training (exercise routine and gait exercises)	Immediately after the intervention	①⑤	No
van Groenestijn et al. (2019) (36)	*n* = 27 Age = 60.9 ± 10.0 F% = 33.0% Netherlands Diagnosis based on the revised El-Escorial criteria	*n* = 27 Age = 59.9 ± 10.7 F% = 27.0% Netherlands Diagnosis based on the revised El-Escorial criteria	16 weeks of aerobic exercise (50–75% HRR, 4 times/week, 30 min/time) + strength exercises (40–50% 1RM, 20 min)	Neuro-palliative care	Immediately after the intervention	①②⑤	No
Ferri et al. (2019) (28)	*n* = 8 Age = 50.7 ± 3.3 F% = 25.0% Australia Diagnosis based on the revised El-Escorial criteria	*n* = 8 Age = 55.5 ± 6.0 F% = 25.0% Australia Diagnosis based on the revised El-Escorial criteria	12 weeks of exercise (3 times/week, 60 min/time, 15 min cardio + 20 min resistance + 10 min balance + 10 min stretching exercises)	Daily activities	Immediately after the intervention	①	No
Kalron et al. (2021) (27)	*n* = 14 Age = 58.5 ± 13.2 F% = 61.1% Israeli Diagnosis based on the revised El-Escorial criteria	*n* = 14 Age = 56.7 ± 11.8 F% = 42.9% Israeli Diagnosis based on the revised El-Escorial criteria	12 weeks aerobic exercise (40–60% HRR, 20-30 min) + passive stretching (10 min) + self-weight exercises (20 min), 2 times/week for 50-60 min	Passive stretching activities (5 times/week, 20 min/time)	Immediately after the intervention	①②③④⑤⑦	No

*n*, sample size; F%, proportion of females; TG, test group; CG, control group; N/A, not reported or unclear; ① = ALSFRS (ALS Functional Scale)/FIM (Functional Independence Measure); ② = FSS (Fatigue Severity Scale)/CIS-Fatigue (Checklist Individual Strength-Fatigue); ③ = WT (Walk Test); ④ = MIP (maximum inspiratory pressure); ⑤ = FVC (forced vital capacity); ⑥ = PEF (peak expiratory flow rate); ⑦ = MEP (maximum expiratory pressure); IMT, inspiratory muscle training; EMST, expiratory muscle training; HRmax, maximum heart rate; SNIP, sniff nasal inspiratory pressure; MIP, maximum inspiratory pressure; MEP, maximum expiratory pressure; MPO, maximum power output. CPET, cardio pulmonary exercise testing; HRR, heart rate reserve; 1RM, 1 repetition maximum force.

### 3.3 Quality assessment results

The majority of included studies reported blinded methods, there was no selective reporting of study results, and it is unclear whether other biases existed. However, most studies did not report methods of randomization and allocation concealment, and most studies had ALS patient dropouts. The primary reason for ALS patients to discontinue exercise is due to physical discomfort caused by disease progression. ALS is a progressive neurodegenerative disease. As the condition worsens, patients experience increasing muscle weakness and atrophy, which may rapidly render them unable to perform exercises they were previously capable of. Moreover, exercise increases the body’s demand for oxygen. Given that patients may have insufficient respiratory function, they could experience dyspnea and shortness of breath during exercise. These symptoms can severely impact the exercise experience and even pose a threat to life, thereby forcing them to stop exercising. The results of the quality assessment of the included studies are shown in Table 2.

**Table 2:** Results of the quality assessment of the included studies.

Included studies	Random methods	Blinding	Allocation concealment	Completeness of outcome data	Selective reporting of findings	Other biases
Bello-Haas et al. (2007) (31)	Unclear	Single blind	Sealed envelope	9 cases withdrawn	No	Unclear
Drory et al. (2001) (26)	Unclear	Unclear	Unclear	7 cases withdrawn	No	Unclear
Merico et al. (2018) (32)	Unclear	Single blind	Unclear	Complete	No	Unclear
Cheah et al. (2009) (29)	Arrange the arrays randomly	Double blind	Sealed envelope	1 cases withdrawn	No	Unclear
Pinto et al. (2012) (34)	Unclear	Double blind	Unclear	4 cases withdrawn	No	Unclear
Plowman et al. (2016) (30)	Unclear	Single blind	Unclear	4 cases withdrawn	No	Unclear
Plowman et al. (2019) (35)	Arrange the arrays randomly	Double blind	Unclear	2 cases withdrawn	No	Unclear
Lunetta et al. (2016) (33)	Unclear	Single blind	Unclear	4 cases withdrawn	No	Unclear
Braga et al. (2018) (11)	Arrange the arrays randomly	Single blind	Unclear	Complete	No	Unclear
van Groenestijn et al. (2019) (36)	Minimize Random	Single blind	Unclear	17 cases withdrawn	No	Unclear
Ferri et al. (2019) (28)	Unclear	Unclear	Unclear	5 cases withdrawn	No	Unclear
Kalron et al. (2021) (27)	Unclear	Single blind	Sealed envelope	4 cases withdrawn	No	Unclear

### 3.4 Meta-analysis results

The results of the heterogeneity test (Table 3) showed that there was a large heterogeneity in ALS function, fatigue, FVC and PEF (*I^2^* > 50%, *p* < 0.1), so the random effects model was used for the main effects test; WT, MIP and MEP had lower heterogeneity (*I^2^* < 50%, *p* > 0.1), so the fixed effects model was used for the main effects test. The results of the main effects test (Table 3) showed that the exercise intervention significantly improved the function of ALS patients [SMD = 0.499, 95% CI = (0.055, 0.943), *Z* = 2.20, *p* = 0.028], increased the distance of WT [SMD = 0.967, 95% CI = (0.448, 1.487), *Z* = 3.65, *p* = 0.000], and increased MEP [SMD = 0.500, 95% CI = (0.043, 0.957), *Z* = 2.14, *p* = 0.032]. However, for fatigue, MIP, FVC, and PEF, the effects of exercise intervention were poor (*p* > 0.05). The meta-analysis results of exercise intervention for ALS are detailed in Supplementary material 1.

**Table 3:** Meta-analysis of the effects of exercise intervention for ALS.

Outcome variables	*N*	Heterogeneity test		Main effects test			
		*I^2^*	*p*	*Z*	*p*	SMD	95% CI
ALS Function	11	77.0%	0.000	2.20	0.028	0.499	(0.055, 0.943)
Fatigue	6	92.1%	0.000	0.72	0.474	0.412	(−0.717, 1.542)
WT	2	11.0%	0.289	3.65	0.000	0.967	(0.448, 1.487)
MIP	3	24.1%	0.268	0.74	0.461	0.175	(−0.290, 0.639)
MEP	2	0.0%	0.850	2.14	0.032	0.500	(0.043, 0.957)
FVC	7	80.3%	0.000	1.19	0.233	0.337	(−0.216, 0.889)
PEF	2	57.7%	0.124	0.58	0.565	0.216	(−0.519, 0.950)

*N* indicates the number of studies included; ALS is amyotrophic lateral sclerosis; WT is the walk test; MIP is maximum inspiratory pressure; MEP is maximum expiratory pressure; FVC is forced vital capacity; PEF is peak expiratory flow rate.

### 3.5 Subgroup analysis

Due to the relative paucity of included studies in WT, MIP, MEP and PEF, subgroup analyses were not invariably performed, so this study investigated the effect of different exercise types on the intervention of ALS using ALS function, fatigue and FVC as dependent variables and exercise type as independent variables, respectively. The results of the heterogeneity test (Table 4) showed that there was a large heterogeneity in the studies of combined exercise interventions for ALS function (*I^2^* > 50%, *p* < 0.1), which were analyzed using a random effects model; the remaining studies of exercise intervention outcome variables had a lower heterogeneity (*I^2^* < 50%, *p* > 0.1), so they were all analyzed using a fixed effects model. Both aerobic and resistance exercise were able to significantly improve the function of ALS patients (*p* < 0.05), with resistance exercise having the largest effect size (*SMD* = 0.686), while combined exercise and respiratory muscle training were not effective interventions for the function of ALS patients (*p* > 0.05). The effects of resistance exercise, combined exercise, and respiratory muscle training on alleviating fatigue in ALS patients were not significant (*p* > 0.05). Aerobic exercise significantly improved FVC in ALS patients (*p* < 0.05), while resistance exercise, combined exercise and respiratory muscle training were not effective interventions for FVC in ALS patients (*p* > 0.05). The subgroup analysis results of exercise intervention for ALS are detailed in Supplementary material 2.

**Table 4:** Subgroup analysis of the effect of different exercise types on ALS.

Outcome variables	Subgroup	*N*	Heterogeneity test		Main effects test			
			*I^2^*	*p*	*Z*	*p*	SMD	95% CI
ALS Function	Aerobic exercise	1	—	—	2.13	0.034	0.629	(0.049, 1.209)
	Resistance exercise	3	0.0%	0.696	3.47	0.001	0.686	(0.299, 1.073)
	Combined exercise	4	91.1%	0.000	1.82	0.069	1.335	(−0.105, 2.776)
	Respiratory muscle training	3	0.0%	0.626	0.22	0.826	−0.042	(−0.414, 0.330)
Fatigue	Resistance exercise	2	52.1%	0.149	1.21	0.225	−0.515	(−1.345, 0.316)
	Combined exercise	3	96.0%	0.000	1.27	0.204	1.485	(−1.808, 3.779)
	Respiratory muscle training	1	—	—	1.56	0.119	−0.627	(−1.416, 0.162)
FVC	Aerobic exercise	1	—	—	5.54	0.000	1.961	(1.268, 2.654)
	Resistance exercise	1	—	—	1.41	0.158	0.368	(−0.143, 0.878)
	Combined exercise	2	0.0%	0.344	1.14	0.256	−0.248	(−0.677, 0.180)
	Respiratory muscle training	3	0.0%	0.828	0.54	0.590	0.112	(−0.295, 0.519)

*N* indicates the number of studies included; ALS is amyotrophic lateral sclerosis; FVC is forced vital capacity.

### 3.6 Sources of heterogeneity

The results of the heterogeneity test showed that there is still a large heterogeneity among the included studies, so the sources of heterogeneity among the included studies remain to be explored. In this study, the effect size of ALS function was used as the dependent variable, and the study characteristics such as publication date, sample size, age of subjects, proportion of females, nationality of subjects, types of exercise, intervention period, frequency of intervention, and duration of exercise were coded as independent variables, and the sources of heterogeneity were explored by univariate meta-regression analysis. The results (Table 5) show that the underlying characteristic variables of the included studies were not a source of heterogeneity between studies of ALS function (*p* > 0.05), and the age of the subjects (*t* = −0.23, *p* = 0.051) reached borderline significant effects and may be a source of heterogeneity between included studies. However, more heterogeneity is not the result of a single factor, but may be the result of multiple factors acting together.

**Table 5:** Results of one-way meta-regression analysis.

Study characteristics	*β*	SE	*t*	*p*	95% CI
Publication date	−0.023	0.055	−0.04	0.689	(−0.148, 0.102)
Sample size	−0.053	0.044	−0.12	0.262	(−0.154, 0.047)
Age of subjects	−0.394	0.171	−2.3	0.051	(−0.788, 0.001)
Proportion of females	0.001	0.037	0.04	0.972	(−0.084, 0.087)
Nationality of subjects	0.267	0.366	0.73	0.485	(−0.562, 1.096)
Types of exercise	−0.270	0.259	−1.04	0.325	(−0.856, 0.317)
Intervention period	0.015	0.024	0.64	0.541	(−0.039, 0.069)
Frequency of intervention	−0.000	0.082	−0.00	0.997	(−0.186, 0.185)
Duration of exercise	0.022	0.022	1.00	0.352	(−0.031, 0.076)

### 3.7 Publication bias test

The reliability of the results of the meta-analysis depends on the presence of bias in the included studies. This study used Egger linear regression for publication bias test. Egger linear regression is a quantitative test for the presence of publication bias to compensate for the lack of subjective judgments in funnel plots (37). The Egger linear regression model constructs a linear regression equation with the effect size as the dependent variable and the precision of the effect estimate as the independent variable, and the intercept of the regression equation is the offset, and the closer it is to 0, the less likely there is publication bias, and if *p* > 0.05 and the 95% CI contains 0, then there is no publication bias (37). Given the relatively few studies included in WT, MIP, MEP, and PEF, it was inconvenient to perform publication bias tests, so only Egger linear regression analyses were performed for ALS function, fatigue, and FVC. The results (Table 6) showed that *p* < 0.05 and 95% CI included 0 for ALS function, indicating the possibility of publication bias in the included studies; fatigue and FVC *p* > 0.05 and 95% CI included 0, indicating the absence of publication bias in the included studies.

**Table 6:** Egger linear regression publication bias test.

Outcome variables	*β*	*SE*	*t*	*p*	95% CI
ALS Function	5.317	1.287	4.13	0.003	(2.406, 8.227)
Fatigue	6.444	5.581	1.15	0.313	(−0.905, 21.939)
FVC	3.022	4.624	0.65	0.542	(−8.864, 14.907)

ALS is amyotrophic lateral sclerosis; FVC is forced vital capacity.

### 3.8 Sensitivity analysis

Sensitivity analysis is an important method used in meta-analysis to assess the robustness and reliability of the combined results and to assess whether the combined results have been significantly changed by the influence of a particular study. In this study, a combined effects test was performed by excluding each study with the help of the “metainf” command, and the estimated range of the SMD for ALS function was found to be −0.256 ~ 10.629, the estimated range of the lower 95% CI was −0.898 ~ 6.572, and the estimated range of the upper 95% CI was 0.202 ~ 14.686, so there may be sensitivity issues with inclusion in the study. In this study, by retrospectively included studies, the studies that caused instability in the combined results were found to be Plowman and Watts et al. (30), Plowman and Tabor-Gray et al. (35) and van Groenestijn et al. (36). After excluding three papers, the results of the combined effects test were similar to those of the main effects test and the results were relatively stable.

## 4 Discussion

The results of the meta-analysis indicate that exercise intervention can improve the function of patients with ALS, increasing the distance of the WT and the maximum MEP. However, exercise intervention is not effective for MIP, FVC, PEF, and fatigue. Further subgroup analysis showed that resistance exercise was the most effective intervention for function in ALS patients, and aerobic exercise was the most effective intervention for FVC in ALS patients. The results of this study are similar to those of the systematic review and meta-analysis by Lui and Byl (38) and Meng et al. (21), both of which support the beneficial effects of moderate exercise in ALS patients. Quality grading of evidence in evidence-based medicine (39) suggests that meta-analysis based on RCTs is the highest level of evidence in clinical practice guidelines. In contrast, the study by Lui and Byl (38) was limited by the paucity of original studies and the inclusion of both animal models and human subjects for analysis, which somewhat reduced the accuracy of the findings. Therefore, researchers have also called for the need for a large number of RCTs to explore the effects of exercise interventions in ALS patients.

The results of the subgroup analysis showed that both aerobic and resistance exercise had a significant effect on function in ALS patients, with resistance exercise having the best effect. While Meng et al. (21) concluded that aerobic exercise had the best effect on ALS patients. Meng et al. (21) included only seven RCTs for combined effects tests and only one study with aerobic and resistance exercise interventions, respectively. Single-study comparisons may ignore the influence of demographic characteristics such as age, gender and duration of illness in the original study, creating a degree of uncertainty in the findings. Clawson et al. (40) explained why resistance training intervention is superior to aerobic exercise. This study evaluated the safety and tolerability of aerobic and resistance exercise in people with ALS and showed that both exercises are safe and tolerable for people with ALS. At the same time, the study noted that resistance exercise showed higher adherence and therefore more effective intervention time in the 24-week intervention, and therefore its intervention was most effective.

The motor neuron lesions caused by ALS lead to atrophy and functional decline of skeletal muscles, including respiratory muscles, making mobility difficult and often feeling fatigue, weakness and low energy (29, 34, 41). Respiratory exercise has been reported to have a beneficial effect on motor neuron disease (42). Clawson et al. (40) also demonstrated that aerobic and resistance exercise is safe and tolerable for ALS patients and does not increase fatigue in ALS patients. Therefore, a large number of studies have focused on investigating the effects of exercise interventions on physical and respiratory function in ALS patients, as well as analyzing the safety and tolerability of exercise by monitoring fatigue after exercise interventions. Meta- analysis results showed that exercise interventions helped to improve physical function and increase walking distances in ALS patients, with more prominent improvements in resistance exercise and aerobic exercise in particular. In addition, Meng et al. (21) showed that ALS patients did not further increase fatigue due to exercise interventions. Our study found that exercise intervention does not increase fatigue in ALS patients, further supporting that exercise therapy is feasible, safe, and well-tolerated by patients with ALS.

Fatigue is a common and potentially debilitating symptom in ALS patients, and subjective measures of fatigue are significantly and negatively correlated with patients’ quality of life (43, 44). Fatigue is mainly caused by ALS or its complications (respiratory failure, speech disorders, malnutrition, etc.) and is exacerbated by factors such as sleep disturbances, depression and anxiety, lack of activity, physical pain and mental disorders (26, 45). Studies related to exercise interventions in neurological disorders (46–48) have shown that moderate exercise can increase muscle mass and muscle strength, improve physiological function, enhance the ability to perform daily living activities and delay disease progression in patients. In addition, exercise interventions have positive effects on improving sleep disorders (49), depression and anxiety (50), and physical pain (51). Based on the above evidence, this study concludes that exercise interventions not only do not exacerbate fatigue in ALS patients, but may also significantly improve quality of life. The results are certainly encouraging and provide an evidence-based basis for promoting improvements in physical function through exercise therapy for ALS patients.

For ALS patients, normal respiratory function is reduced or even lost due to atrophy of the external intercostal muscles involved in breathing, as well as stiffness of the thoracic joints, which limits respiratory movements (52). Improving respiratory impairment in ALS patients through exercise can help improve the quality of survival, but the results of related studies are more controversial. A meta-analysis by Rahmati and Malakoutinia (20) showed that aerobic exercise, resistance exercise and combined exercise were not effective in improving respiratory function in ALS patients. A meta-analysis by Meng et al. (21) showed that exercise can improve FVC in ALS patients. In a meta-analysis by Ferreira et al. (42), which included both multiple sclerosis (MS) and ALS patients, a combined effects test showed that MEP and MIP were improved by respiratory muscle training in both MS and ALS patients, but the effect of the intervention was not significant for FVC in MS and ALS patients. The results of this study showed that exercise intervention helped to promote the improvement of MEP in ALS patients, but was not significant for MIP, FVC and PEF. In addition, for the first time, aerobic exercise was shown to be effective in promoting the improvement of FVC in ALS patients through subgroup analysis. Previous studies (53, 54) have shown that diaphragmatic pacing facilitated by exercise increases the amplitude of diaphragmatic movement, increases muscle thickness, improves ventilation and delays the decline in FVC. However, in ALS patients with restricted mobility, diaphragmatic pacing can cause venous thrombosis (53, 54). Eidenberger et al. (55) also showed that the evidence for improving respiratory function in ALS patients through respiratory muscle training is still insufficient. The relative paucity of studies included in this study and the large heterogeneity of some of the indicators also make it difficult to demonstrate the positive effects of exercise interventions on respiratory function in ALS patients.

Skeletal muscle is an important tissue involved in the development of ALS disease through the activation of retrograde signaling cascades that degrade motor neurons (56). The health and function of skeletal muscle is influenced by satellite cells, mitochondria and micoRNA (56). Based on the role of skeletal muscle in the development of ALS, it can be hypothesized that exercise improves muscle function and delays muscle decay by improving motor neurons. Metallothioneins (MTs) are strong scavengers of reactive oxygen species and have some neurotropic activity, so exercise may have a beneficial effect on spinal cord motor neurons in ALS patients due to the induction of MTs (52). Hashimoto et al. (57) showed that regular exercise on a treadmill increased the mRNA expression levels of MTs in mice and promoted the accumulation of MTs in the spinal cord, while promoting an enhanced immune response to MTs in astrocytes. Kassa et al. (58) also showed a potential neuroprotective effect of exercise in ALS patients, with exercise significantly improving microglia activation when damage to motor neurons was less severe. In addition, Lei and Huang (2) suggested that moderate exercise has a protective effect on motor neurons in ALS patients and can improve muscle function and disease progression in ALS patients through the expression of glucose transporter protein and glyceraldehyde-3-phosphate dehydrogenase.

Based on the key findings of this study, it is recommended that a comprehensive and effective treatment plan for ALS patients can be provided by integrating personalized exercise programs with professional multidisciplinary support. Firstly, ALS patients are advised to engage in moderate exercise and select the appropriate type of exercise according to their actual conditions, aiming to enhance their overall quality of life and physical capabilities. Secondly, when formulating an exercise plan, it is essential to consider the patient’s specific health status and functional level to ensure the safety and suitability of the exercises, to prevent exercise-related injuries, and to ensure that the exercise does not exacerbate the patient’s sense of fatigue. Lastly, this study encourages collaboration among multidisciplinary teams to provide education on exercise interventions to patients and their families, helping them understand the benefits and potential risks associated with exercise, and offering psychological support to assist patients in dealing with the emotional and psychological challenges posed by ALS.

However, this study still has the following limitations. Firstly, most of the included studies did not explicitly report the methods of randomization and measures of allocation concealment, and most of the studies had subject dropouts, presenting a potential risk of bias. Secondly, the research on ALS function was affected by publication bias and sensitivity issues. Thirdly, there were relatively few studies included regarding WT, MIP, MEP, and PEF, thus further exploration of the impact of exercise intervention on ALS is warranted. Finally, due to the limitations in the number of original studies, this study has only conducted subgroup analyses based on exercise type and has not yet analyzed other factors such as intervention duration, duration of each intervention session, and participant demographics. Therefore, it is not possible to provide more precise evidence at this stage. We hope that future studies will expand the pool of original research and further supplement the evidence for exercise interventions in ALS.

## 5 Conclusion

Exercise interventions can improve function, increase WT distance and MEP in ALS patients. Of these, resistance exercise was the most effective intervention for function in ALS patients, and aerobic exercise was the most effective intervention for FVC in ALS patients. This study recommends that a comprehensive and effective treatment plan for ALS patients can be provided by integrating personalized exercise programs with professional multidisciplinary support. However, due to the small number of included studies and possible issues of heterogeneity, risk of bias and sensitivity, the effects of the exercise intervention ALS need to be further explored.

## Data Availability

The raw data supporting the conclusions of this article will be made available by the authors, without undue reservation.
