# Supplementary material for: The effect of exercise intervention on amyotrophic lateral sclerosis: a systematic review and meta-analysis
Source: Front Neurol. 2025 May 21;16:1499407. doi: 10.3389/fneur.2025.1499407 (PMC12133518; doi:10.3389/fneur.2025.1499407)
Supplement: Supplementary file 1 [file Table_1.docx]

# Supplementary Material 1: Meta-analysis of the effects of exercise intervention for ALS


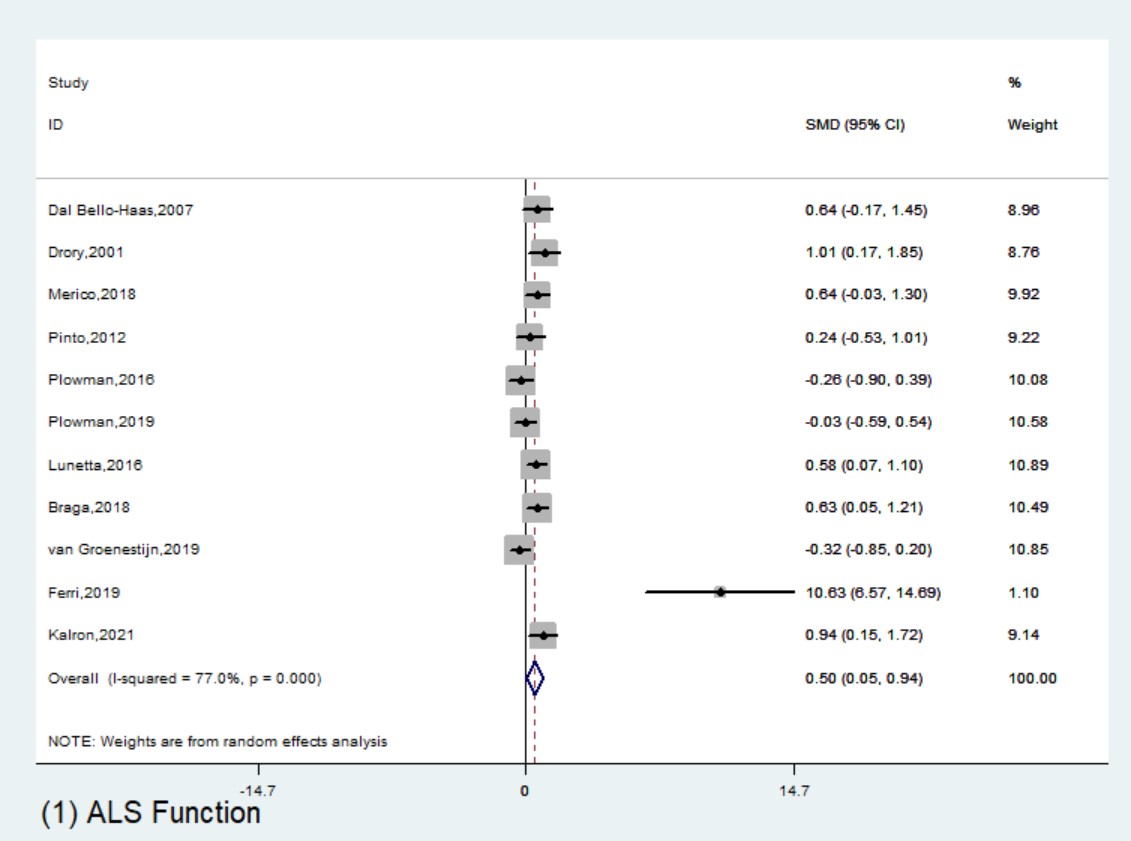


**Figure 1. The forest plot of the functional effects of exercise intervention in ALS**


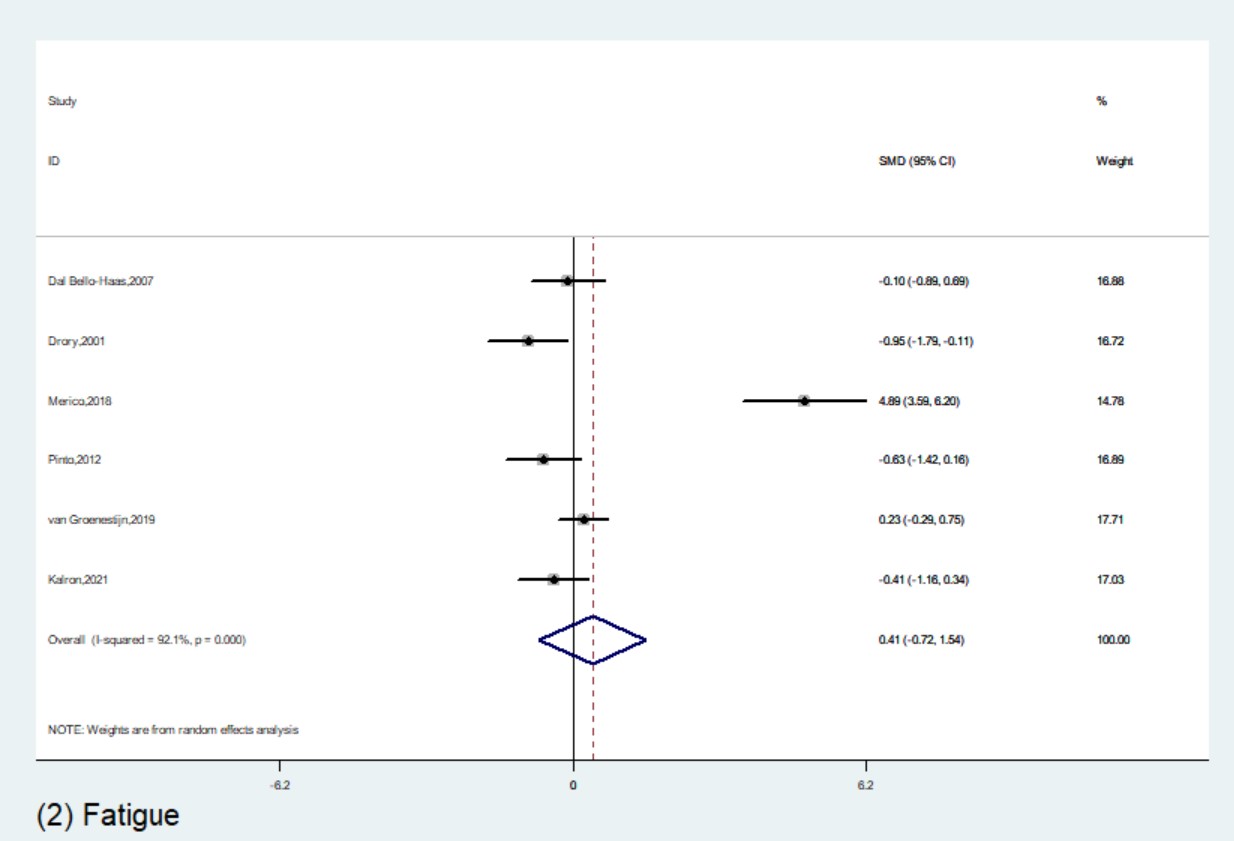


**Figure 2. The forest plot of the functional effects of exercise intervention in fatigue**


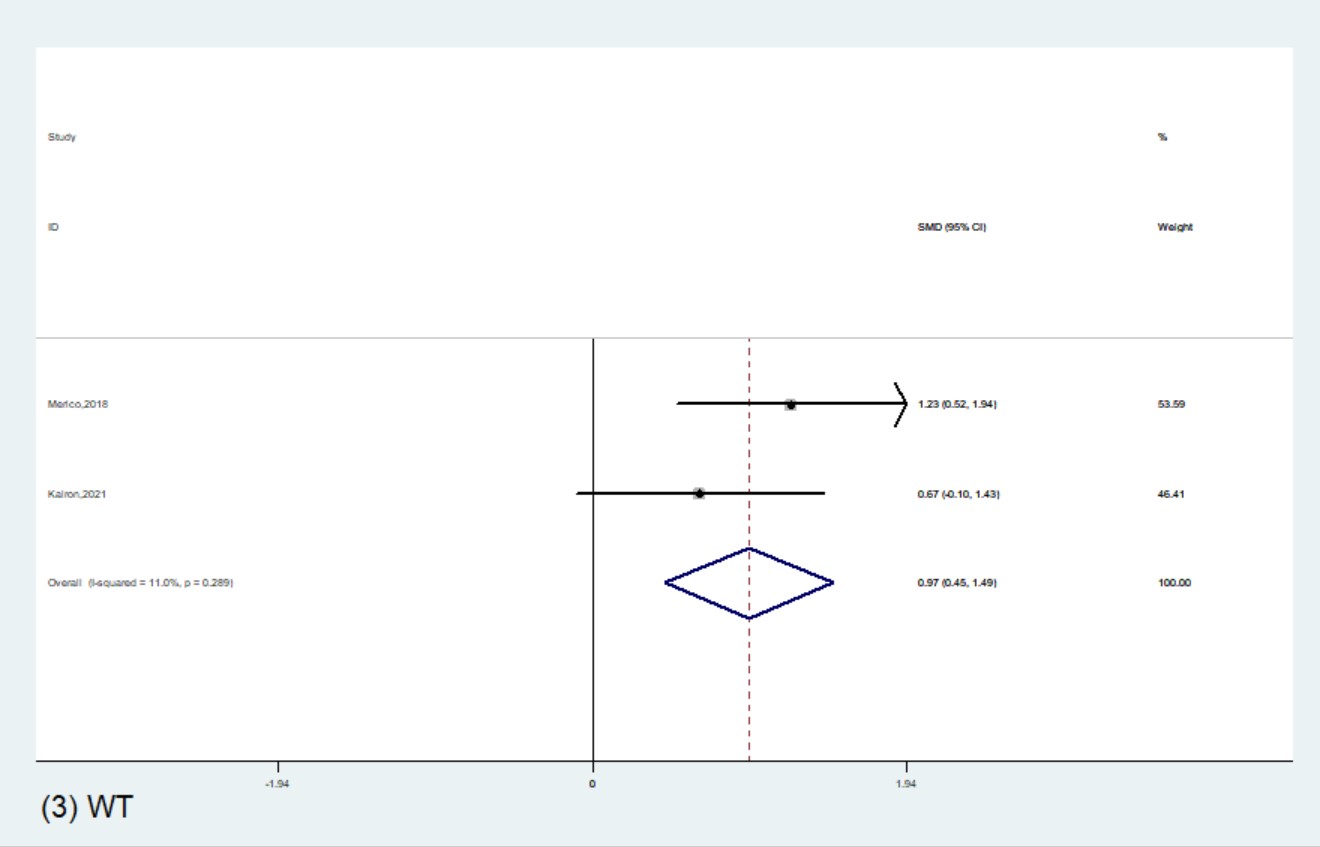


**Figure 3. The forest plot of the functional effects of exercise intervention in WT**


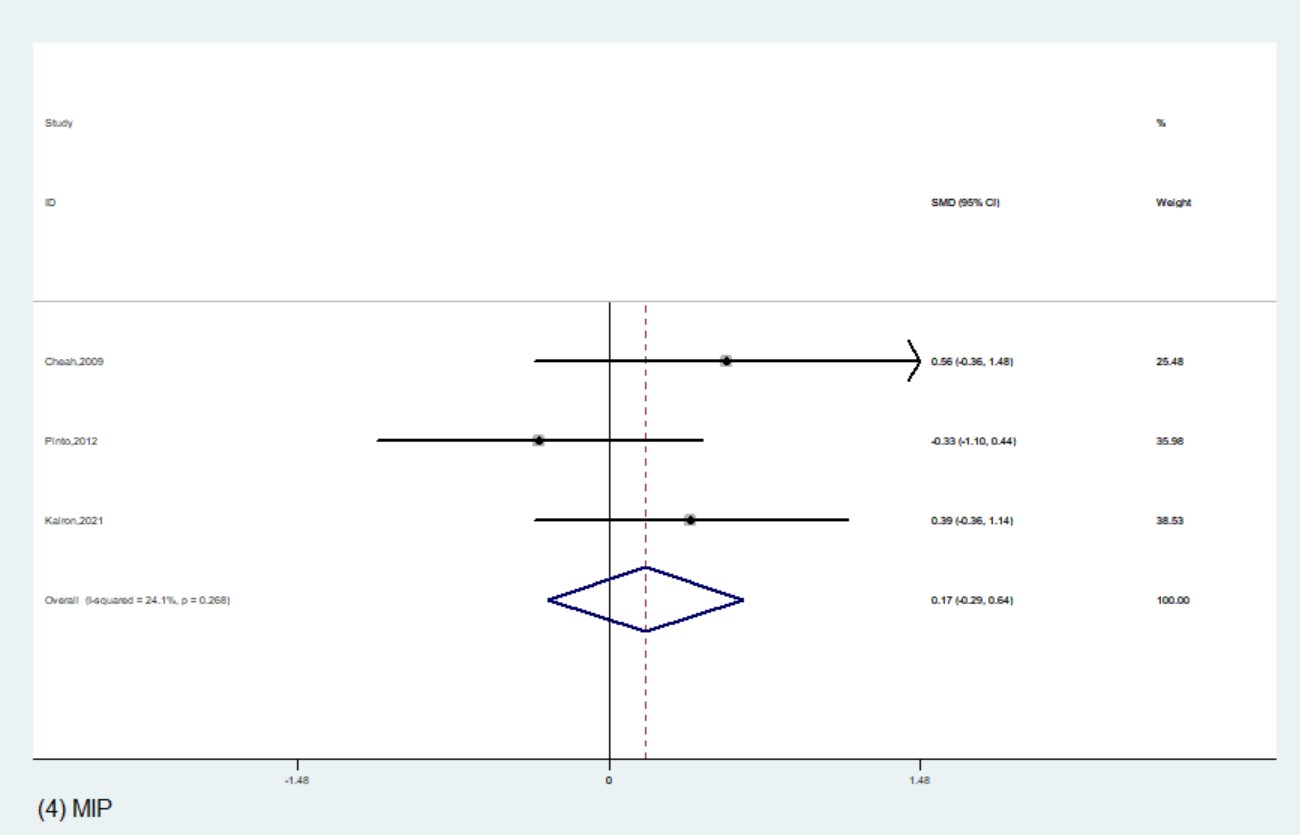


**Figure 4. The forest plot of the functional effects of exercise intervention in MIP**


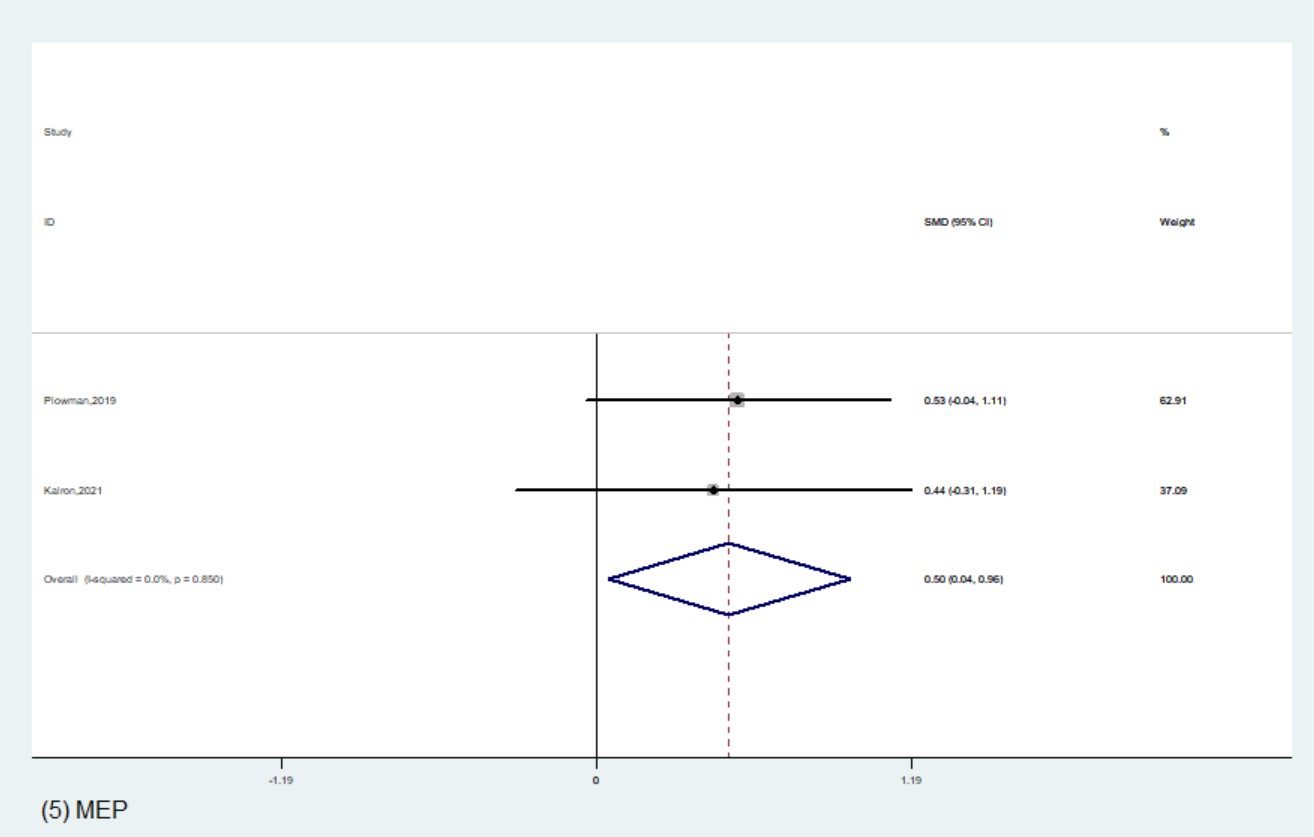


**Figure 5. The forest plot of the functional effects of exercise intervention in MEP**


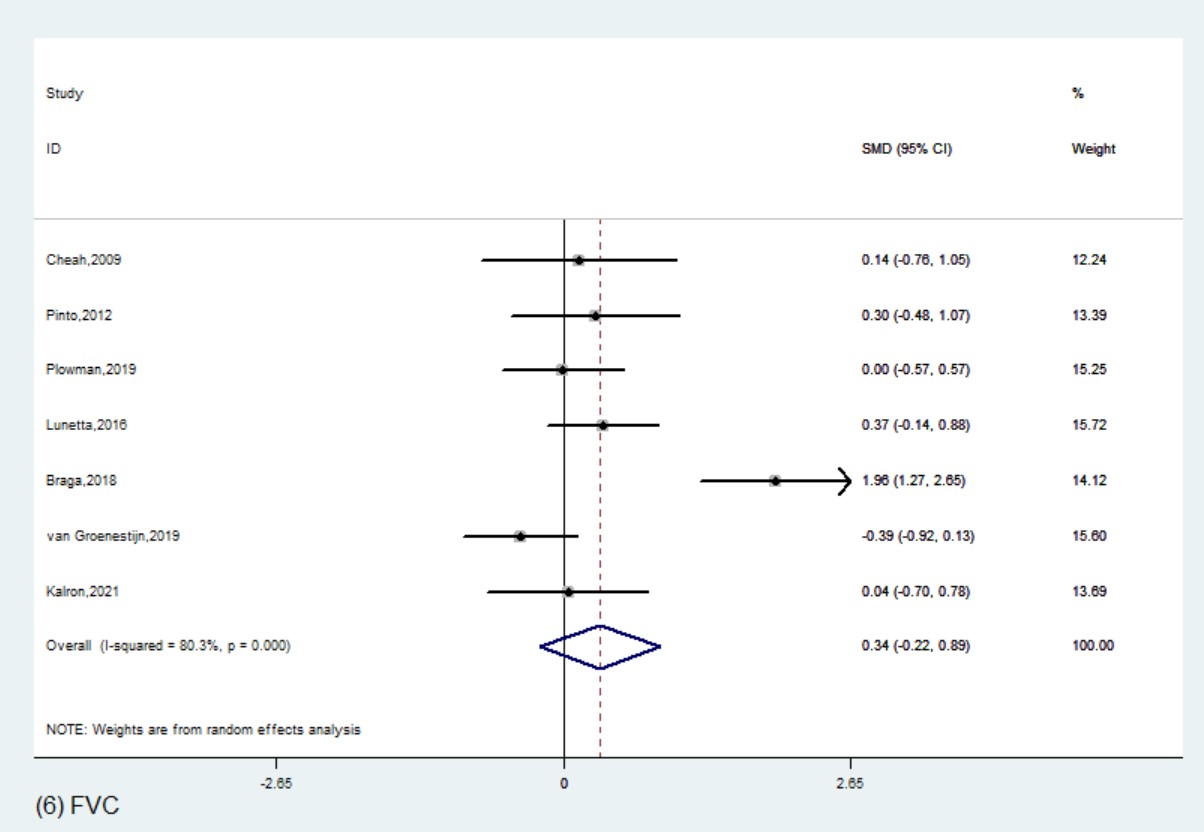


**Figure 6. The forest plot of the functional effects of exercise intervention in FVC**


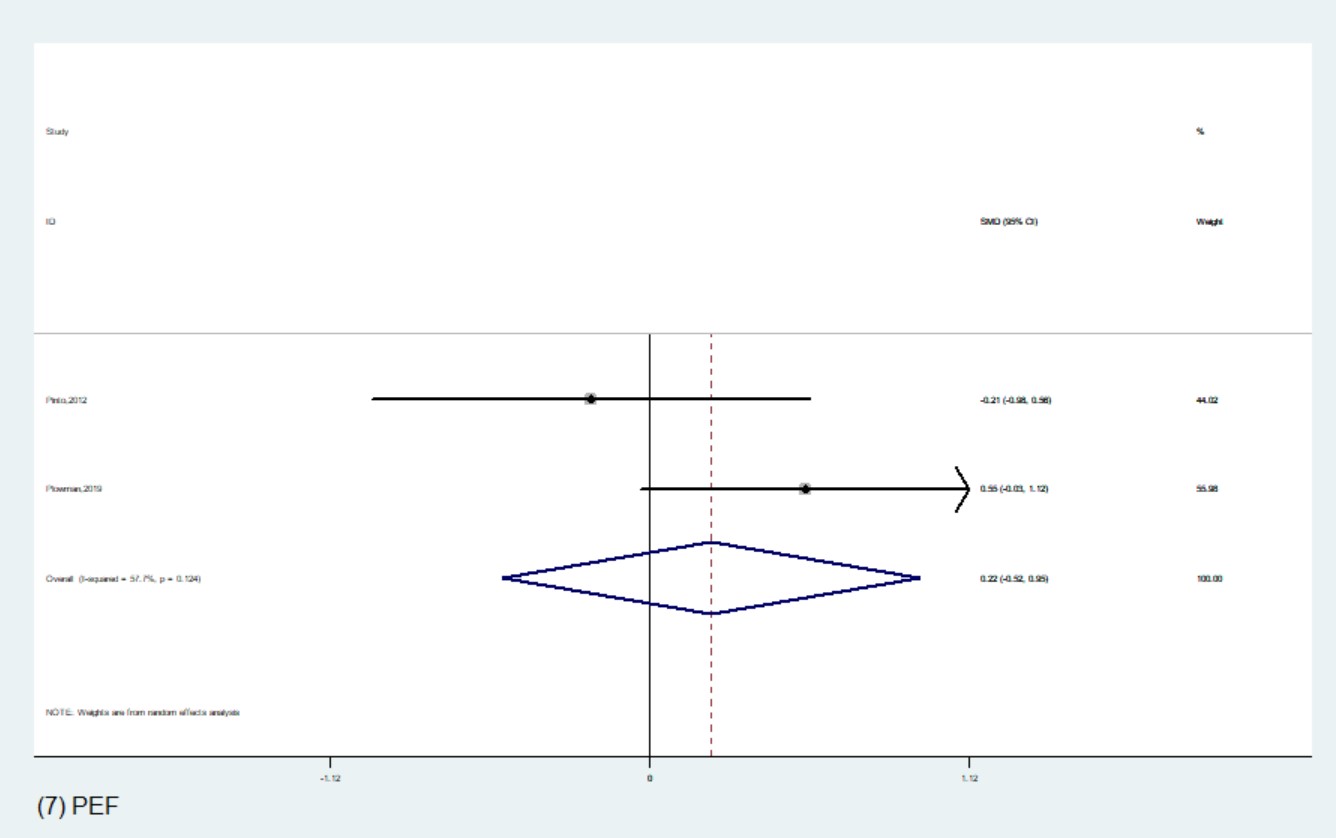


**Figure 7. The forest plot of the functional effects of exercise intervention in PEF**
