# Supplementary material for: The effect of exercise intervention on amyotrophic lateral sclerosis: a systematic review and meta-analysis
Source: Front Neurol. 2025 May 21;16:1499407. doi: 10.3389/fneur.2025.1499407 (PMC12133518; doi:10.3389/fneur.2025.1499407)
Supplement: Supplementary file 2 [file Table_2.docx]

# Supplementary Material 2: Subgroup analysis of the effect of different exercise types on ALS


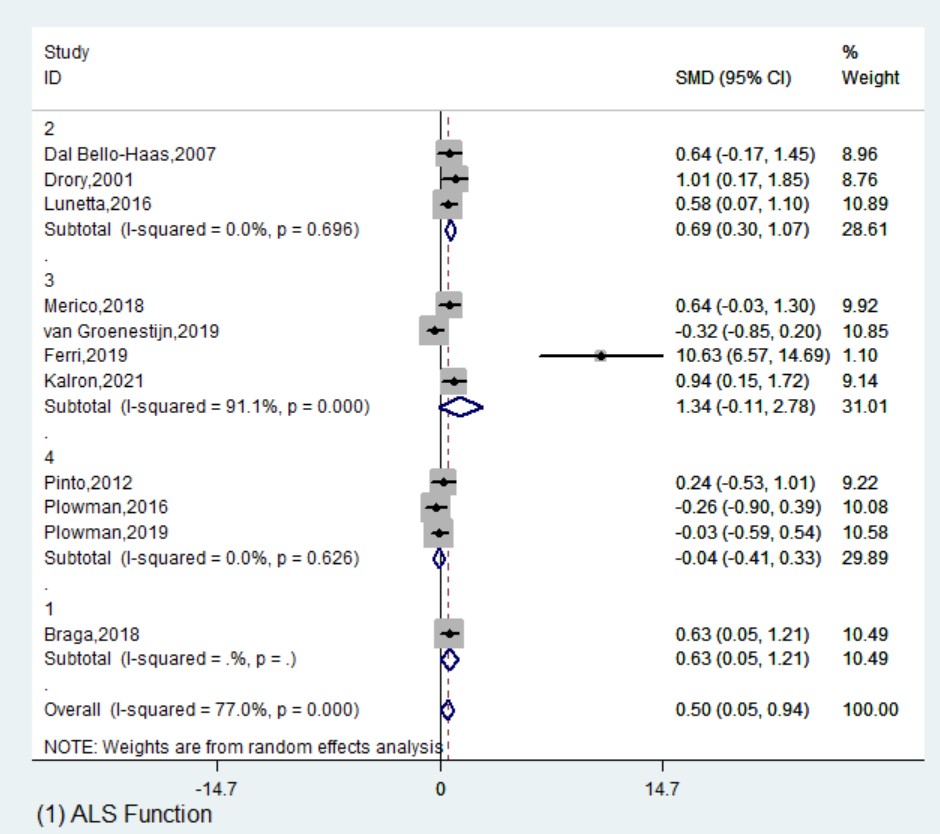


**Figure 1. Subgroup analysis of the functional effects of exercise intervention in ALS (Notes: 1 indicates aerobic exercise; 2 indicates resistance exercise; 3 indicates combined exercise; 4 indicates respiratory muscle training)**


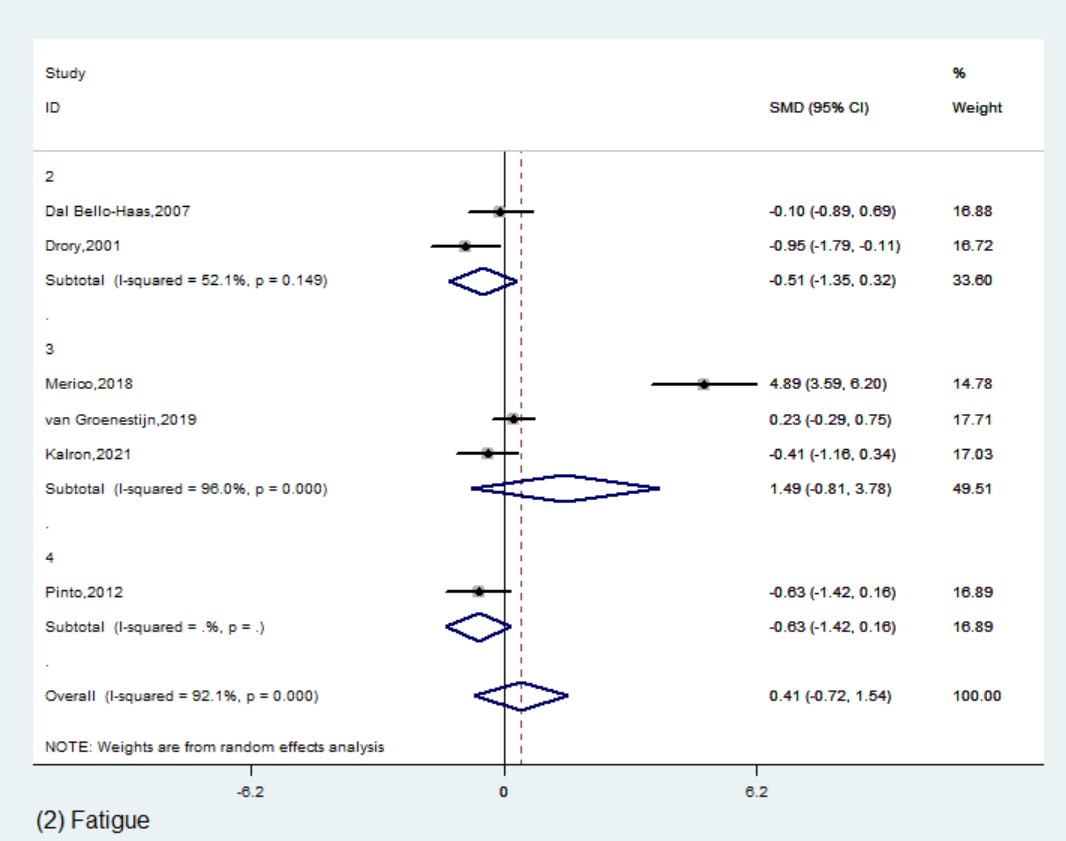


**Figure 2. Subgroup analysis of the functional effects of exercise intervention in fatigue (Notes: 1 indicates aerobic exercise; 2 indicates resistance exercise; 3 indicates combined exercise; 4 indicates respiratory muscle training)**


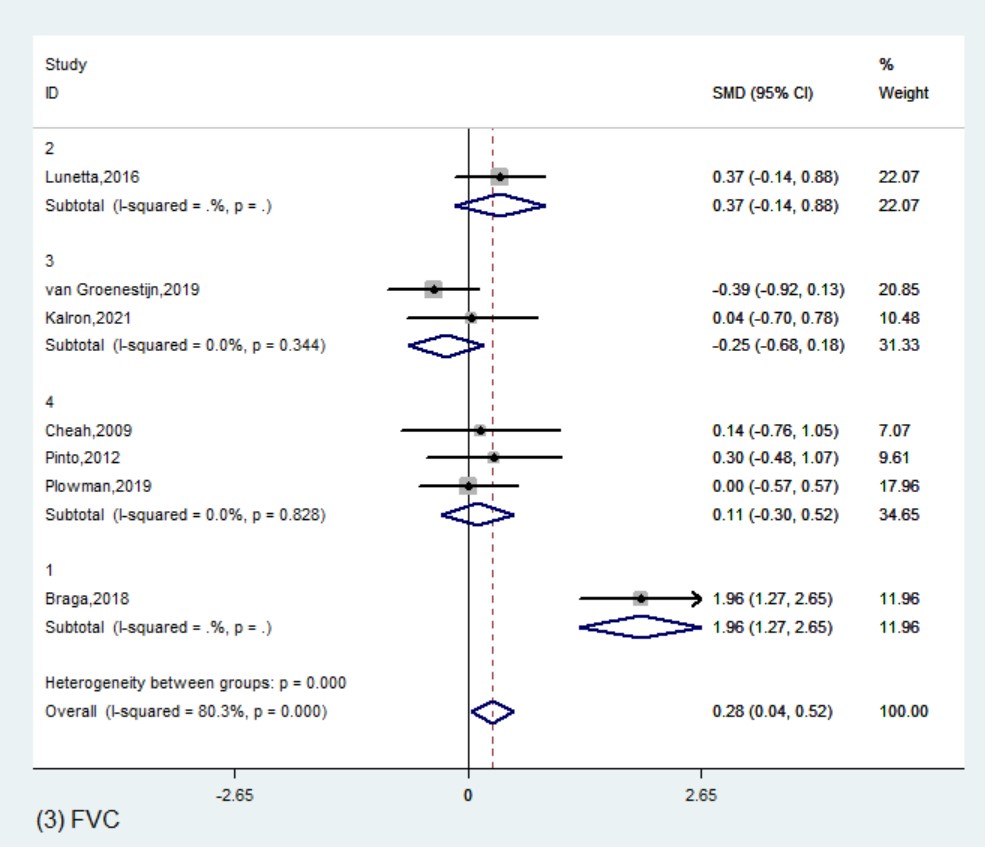


**Figure 3. Subgroup analysis of the functional effects of exercise intervention in FVC (Notes: 1 indicates aerobic exercise; 2 indicates resistance exercise; 3 indicates combined exercise; 4 indicates respiratory muscle training)**
